# Degradation and Regeneration of Soil Structure in Intensified Paddy Fields: Plant–Soil Interactions, Ecological Effects, and Restoration Pathways

**DOI:** 10.3390/plants15142225

**Published:** 2026-07-21

**Authors:** Meng Fang, Jiahao Shen, Gan Liu, Chirui Zhang, Zhong Tang

**Affiliations:** School of Agricultural Engineering, Jiangsu University, Zhenjiang 212013, China

**Keywords:** intensified paddy fields, soil structural degradation, rice root growth, plant–soil interactions, targeted restoration

## Abstract

Intensified paddy production plays a crucial role in sustaining rice productivity and food security; however, long-term high-frequency puddling, heavy machinery operations under wet soil conditions, simplified cropping systems, and insufficient organic matter inputs have progressively degraded the physical structure of paddy soils. Such structural degradation not only weakens soil water movement, nutrient supply, and aeration but also restricts rice root penetration, alters rhizosphere processes, and disrupts plant–soil feedbacks. Previous studies have largely focused on individual aspects such as soil compaction, amendment-based improvement, water management, or root responses, whereas an integrated understanding of the multi-source drivers, functional consequences, and restoration pathways of soil structural degradation in intensified paddy fields remains limited. Following the overarching theme of soil degradation and regeneration, this review systematically synthesizes the indicator framework, formation mechanisms, degradation typology, ecological consequences, and regulation strategies of paddy soil structural degradation. We further clarify the transition of degraded paddy soils from single physical constraints to the coupled decline of physical, chemical, and biological functions, and compare the agronomic performance, environmental implications, implementation feasibility, and trade-offs of different restoration pathways. Existing evidence indicates that soil structural degradation in paddy fields can impair root-zone pore connectivity, rhizosphere oxygen supply, nutrient acquisition, microbial-mediated carbon and nitrogen cycling, and greenhouse gas regulation, thereby affecting rice growth, yield stability, and the ecological sustainability of paddy systems. Accordingly, the restoration of degraded paddy soils should move beyond short-term loosening or single-factor amendment toward integrated regeneration strategies that maintain soil structural health, reconstruct plough-layer functions, enhance root–soil interactions, and promote the synergistic recovery of pore networks, aggregates, organic carbon, and microbial processes. This review provides a theoretical basis and research reference for the precise restoration of soil structural constraints and the sustainable management of plant–soil systems in intensified paddy fields.

## 1. Introduction

Global food security, constraints on arable land resources, and changes in rural labour availability are reshaping paddy production systems, but the pathways of intensification differ substantially among regions [1,2]. In large-scale production areas, intensification is often accompanied by increased machinery use and heavier field operations, whereas in smallholder-dominated regions, it may rely more on labour-saving technologies, improved water and nutrient management, diversified cropping systems, or service-based mechanization. Despite these regional differences, intensified paddy management commonly increases the frequency and intensity of soil disturbance, thereby raising concerns about the long-term stability of paddy soil structure.

While intensified management increases production efficiency, it also substantially alters the structural state that paddy soils have developed over long-term cultivation [3,4]. The combined effects of high-frequency tillage, repeated puddling, machinery traffic under wet soil conditions, alternating long-term flooding and drainage, and insufficient organic matter return have gradually shifted paddy soil structure from a relatively stable hydromorphic plough layer toward compaction, structural dispersion, and functional weakening [5,6]. As the physical foundation linking crop root growth, water movement, aeration, nutrient transformation, and microbial activity, soil structure degradation not only modifies the physical condition of the plough layer but also directly constrains rice root distribution, rhizosphere oxygen supply, nutrient acquisition, and yield stability, thereby weakening both productivity maintenance and ecological functional stability in paddy systems [7,8,9].

Previous studies have shown that agricultural machinery-induced compaction can significantly increase soil bulk density and penetration resistance, reduce porosity, water infiltration capacity, and gas diffusivity, and subsequently affect crop root growth and yield formation [10,11]. Excessive soil compaction reduces water permeability and restricts root elongation, thereby inhibiting crop growth [12]. Mechanical load, soil water content, texture, organic matter content, and traffic frequency jointly determine the degree of soil compaction and associated structural degradation [13]. Nevertheless, structural degradation in paddy fields is not merely a process of physical compaction. Rather, it represents a systemic process involving changes in pore networks, plough-layer configuration, hydrological responses, and ecological functions [14], and is closely associated with organic carbon inputs, aggregate protection, and microbial-mediated carbon and nitrogen transformations [15,16,17].

Extensive research has examined the degradation and improvement of paddy soils, with major interventions including subsoiling and deep tillage, organic material return, biochar amendment, cropping-system optimization, and tillage-system adjustment [18,19,20,21]. Across these studies, a common restoration logic can be identified: physical loosening mainly targets compacted subsurface layers, whereas organic inputs and diversified cropping systems primarily contribute to aggregate rebuilding, pore stabilization, and biological recovery. Straw return, green manure incorporation, and crop rotation increase exogenous carbon inputs and may enhance labile organic carbon, microbial carbon-source utilization, and aggregate-associated carbon accumulation [22,23,24]. From a plant–soil interaction perspective, these practices can be interpreted as a linked pathway. Consistent improvements have been reported in bulk density, porosity, crop yield, and soil organic carbon accumulation [25,26]. However, the magnitude and persistence of these benefits are highly context dependent. Their effectiveness varies with the initial degradation type, soil texture, organic matter status, residue quality, hydrological regime, fertilizer timing, and duration of implementation. Therefore, the same practice may act as a structural amendment in one paddy system but show limited persistence or even cause secondary risks in another.

Water-regime regulation further illustrates this context dependency. Long-term flooding, midseason drainage, alternate wetting and drying (AWD), and controlled irrigation alter pore water occupancy, redox conditions, water fluxes, and nitrogen transport pathways, thereby influencing both soil structural functions and ecological outcomes [24]. Moderate AWD may improve water-use efficiency and reduce methane emissions under suitable thresholds, whereas excessive or poorly timed wet–dry alternation can promote crack development, carbon and nitrogen losses, enhanced nutrient transport, and increased nitrous oxide (N_2_O) emissions [27]. Similarly, biochar amendment may improve soil porosity, water retention, and nutrient retention when combined with AWD or controlled irrigation, but its effects depend strongly on feedstock properties, application rate, soil background, irrigation intensity, and long-term management conditions [28]. Conservation tillage, residue mulching, green manure substitution, and reduced chemical fertilizer input also show potential for improving subsoil strength, pore-size distribution, root growth, aggregate stability, and organic carbon protection, but their outcomes are shaped by local cropping systems and soil–microbe–carbon interactions [29,30,31]. Collectively, existing evidence suggests that paddy soil structural restoration should not be evaluated only by whether a single measure improves one soil property. A more critical framework is needed to assess whether a given practice can match the dominant degradation type, rebuild stable pore networks, maintain rhizosphere function, support rice productivity, and avoid undesirable trade-offs such as recompaction, nutrient loss, or greenhouse gas emissions.

**Figure 1:**
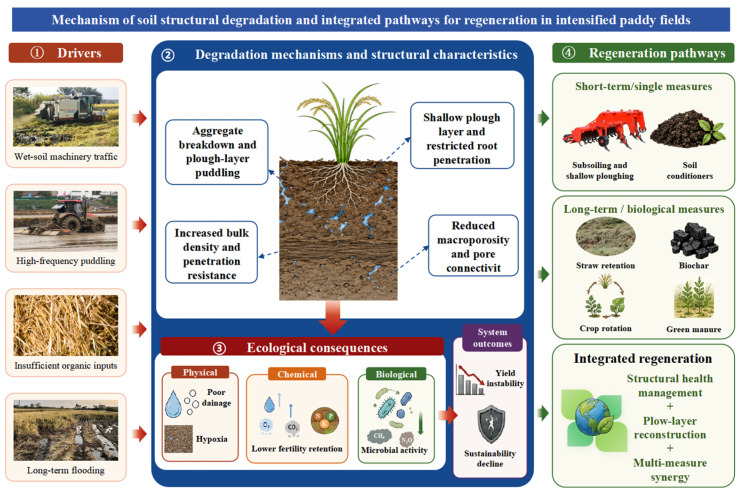
Mechanism of soil structural degradation and integrated pathways for regeneration in intensified paddy fields.

However, existing studies have mostly focused on individual restoration technologies, with limited systematic classification and targeted analysis based on degradation drivers. Process-based indicators such as pore connectivity, water movement, aeration status, and rhizosphere structural reconstruction have received insufficient attention. Moreover, most current studies have not established targeted restoration pathways for different degradation types, resulting in a mismatch between restoration measures and structural constraints. Accordingly, this review focuses on soil structural degradation in intensified paddy fields. Compared with previous reviews that mainly addressed soil compaction, paddy soil fertility decline, or individual restoration measures, this review is designed as a conceptual and synthesis review with an operational restoration framework. Its novelty lies in three aspects. First, soil structural degradation in intensified paddy fields is interpreted as a transition from single physical constraints to coupled physical, chemical, biological, and plant–soil functional decline. Second, a degradation typology is proposed to distinguish compaction-dominated, structure-dispersion-dominated, and compound degradation according to dominant drivers, affected soil layers, structural symptoms, and functional constraints. Third, restoration pathways are evaluated by linking mechanical loosening, organic inputs, water-regime regulation, root-rhizosphere processes, microbial functional groups, greenhouse gas trade-offs, and field applicability. This framework aims to move the discussion from a descriptive summary of practices toward a diagnosis-based and plant–soil-centred restoration strategy. On this basis, we propose a soil structural health management framework for sustainable rice production, aiming to provide a theoretical reference for the remediation of structural constraints and the improvement of cultivated land quality in intensified paddy fields, as illustrated in Figure 1.

## 2. Formation Mechanisms and Major Indicators of Soil Structural Degradation in Intensified Paddy Fields

Soil structure refers to the spatial arrangement of soil solid particles formed through the combined effects of organic matter, clay minerals, biological activity, and external disturbances [32,33]. In agricultural soils, soil structure is not merely a morphological attribute; rather, it provides the fundamental physical framework that regulates water retention and supply, aeration and gas exchange, root penetration, nutrient retention, and the habitat space for microbial activity [34]. In this review, soil structural degradation is used as an umbrella concept referring to the deterioration of soil physical architecture and its associated plant–soil functions. Soil compaction is considered one important form of structural degradation, characterized mainly by increased bulk density, penetration resistance, and mechanical impedance. However, structural degradation is broader than compaction and also includes aggregate breakdown, pore-network disconnection, loss of biopores, surface sealing, and the decline of water–gas transport, rhizosphere resource supply, and root-zone functionality. This distinction is important because compaction-dominated, structure-dispersion-dominated, and compound degradation types require different diagnostic indicators and restoration pathways.

### 2.1. Physical Disruption Caused by Machinery Disturbance and Wet-Soil Trafficking

**Figure 2:**
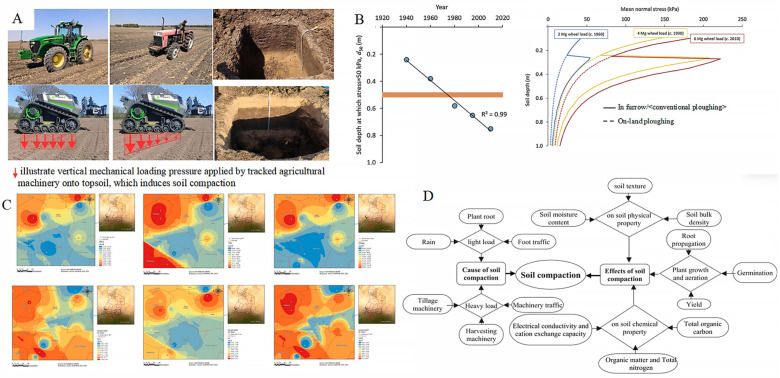
Machinery-induced soil compaction, subsoil stress transmission, and structural-functional degradation in intensified paddy fields. (**A**) Schematic representation of field-scale soil compaction and soil profile responses under tracked and wheeled machinery operations. (**B**) Depth reached by a vertical stress of 50 kPa and the mean normal stress beneath the in-furrow wheel during conventional ploughing. (**C**) Spatial distribution of soil organic carbon, total nitrogen stocks, and carbon dioxide equivalent in the 0–20 cm and 40–60 cm soil layers. (**D**) Conceptual pathway linking soil compaction drivers to structural and functional consequences. Panels (**A**–**D**) were adapted or redrawn based on previous studies [38,41,42,43].

In intensified rice production systems, high-frequency land preparation, repeated puddling, and machinery operations under wet soil conditions represent the most direct external disturbances driving paddy soil structural degradation (Figure 2A). With the rapid development of agricultural mechanization, the mass and wheel or track loads of tractors, combine harvesters, and tracked machinery have increased substantially, thereby enhancing the mechanical stresses transmitted into the soil profile. Under high water content or near-saturated conditions, the shear strength and bearing capacity of paddy soils decline markedly. As a result, mechanical loads are more readily transmitted downward through the tire–soil or track–soil contact interface, leading to stress concentration in the lower plough layer and subsoil [35]. This stress transmission process is not restricted to superficial disturbances; rather, it can extend progressively into deeper soil layers as machinery load and traffic frequency increase. Previous studies have shown that increasing machinery load deepens the soil layer affected by critical vertical stress and increases the mean normal stress imposed on the subsoil under conventional in-furrow ploughing (Figure 2B). The impact of machinery traffic under wet soil conditions should therefore be interpreted not simply as surface puddling or short-term rutting, but as a cumulative structural degradation process driven by surface load input, profile-scale stress transmission, and subsoil reorganization.

Long-term mechanical disturbance and wet trafficking further alter the physical structure and biogeochemical status of paddy soil profiles. As traffic frequency increases, soil dynamic deformation modulus and compactness generally rise rapidly and then approach a stable state, indicating a transition from a deformable soil matrix to a denser structural state under repeated loading [36,37]. At the profile scale, this process is commonly manifested as enhanced penetration resistance at specific depths, the development of a plough pan or compacted layer, increased bulk density, reduced macroporosity, and restricted root penetration. The evidence summarized in Figure 2C further indicates that long-term cultivation not only modifies soil physical structure but also affects the spatial distribution of soil organic carbon, total nitrogen stocks, and carbon dioxide equivalent emissions across different soil depths. Mouaromba Wavel et al. reported significant changes in soil bulk density and organic matter under different cultivation durations, suggesting that long-term land use intensifies both physical and nutrient degradation in soils [38,39]. These findings indicate that mechanical compaction is not an isolated physical process; instead, it may alter pore structure, root distribution, carbon and nitrogen accumulation, and gas transport conditions, thereby inducing broader functional decline in paddy soils.

From a process-chain perspective, machinery-induced soil compaction can be conceptualized as a continuous pathway involving external load input, soil structural deformation, pore-network degradation, restrictions on root growth and water–gas transport, and subsequent declines in production and ecological functions (Figure 2D). As emphasized by Pittelkow et al., the loss of soil physical structure can weaken the ecological resilience of agricultural systems to weather anomalies and extreme climatic stresses, eventually becoming a key structural constraint on water infiltration, deep root growth, and long-term system sustainability [40]. Accordingly, in intensified paddy fields, long-term high-frequency puddling and machinery traffic under wet conditions do not merely alter the surface puddled state. Instead, they promote structural degradation through repeated stress input and continuous subsoil compaction, gradually transforming the soil from a plough layer structure with relatively favourable permeability and buffering capacity into a compacted, occluded, and functionally weakened constraint layer.

Increased bulk density and penetration resistance are the most direct and widely used diagnostic indicators of soil compaction, but they only partially reflect the broader process of structural degradation. Previous studies have shown that enhanced mechanical disturbance generally increases bulk density and penetration resistance in both topsoil and subsoil, with these changes being particularly pronounced under high-intensity management and wet trafficking conditions [42,44,45]. More fundamental structural changes occur through the reorganization of the pore system. A well-connected hierarchical pore network is gradually transformed into a structure dominated by small pores, isolated pores, and poorly connected pore domains, resulting in reduced macroporosity and weakened air permeability and gas diffusivity [46]. Using X-ray computed tomography, Ogorodniaia et al. found that, with increasing compaction intensity, large oriented pores were progressively replaced by smaller isolated pores, and the soil structure tended to become denser and more massive [47]. Longepierre et al. further demonstrated that even a single compaction event can significantly increase soil bulk density while reducing air permeability and gas diffusion capacity, and such effects may remain incompletely recovered over several subsequent growing seasons [48]. Thus, machinery-induced structural degradation is not a single-index response but the combined outcome of particle rearrangement, aggregate disruption, pore collapse, recompaction, and profile deformation [13,49]. In studies of machinery operations in forest and agricultural soils, compaction, soil displacement, and rutting are commonly used as key response variables for assessing mechanical disturbance, with their severity primarily governed by wheel load, traffic frequency, and initial soil water status [50]. Taken together, mechanical compaction in intensified paddy fields should be understood as a profile-scale structural reorganization process driven by the superposition of surface disturbance and subsoil densification. This process progressively restricts water infiltration, gas exchange, rooting space, and root-mediated resource acquisition, thereby weakening both soil structural recovery capacity and the functional stability of rice-based production systems.

### 2.2. Aggregate Disruption and Decline of Biopore Networks

In intensified paddy fields, structural degradation is expressed not only as increased bulk density in the plough layer or enhanced subsoil compaction, but also as aggregate destabilization, biopore loss, and declining pore continuity [51]. These microstructural changes are functionally important because soil structure regulates pore distribution, water–air configuration, microbial habitats, carbon stabilization, and root-zone resource supply [52]. Under repeated puddling, wet-soil machinery operations, and insufficient organic inputs, aggregate disruption and biopore-network decline tend to reinforce each other. Reduced organic return weakens organo-mineral binding and decreases the supply of aggregate-binding agents, while mechanical disturbance breaks down structural units and interrupts continuous pore domains [53,54,55,56]. As a result, the soil may shift from a hierarchical pore system with relatively stable aggregates to a fragmented structure characterized by unstable aggregates, isolated pores, and limited biological recovery capacity.

This process does not necessarily appear as a large increase in bulk density. In many cases, aggregate-scale pore reorganization precedes obvious macroscopic compaction, which helps explain why different studies emphasize different indicators of degradation. For example, SR-μCT evidence shows that plough tillage and subsoiling, especially when combined with straw incorporation into the subsoil, can increase total aggregate porosity and connected porosity, indicating that mechanical loosening and organic input jointly promote pore formation and connectivity [57]. In contrast, studies using organic carbon-to-clay ratios or mineral-associated carbon indicators emphasize the role of carbon protection and mineral binding in maintaining structural stability [58]. For paddy soils, a surface layer may appear temporarily loose after tillage, but if aggregate cohesion, organic binding, and biopore continuity are not restored, the soil remains vulnerable to re-dispersion, pore collapse, and renewed compaction. Thus, aggregate stability and biopore continuity should be evaluated together when diagnosing structural degradation, especially where rice root growth, rhizosphere aeration, and nutrient acquisition depend on the persistence of connected pore networks.

In addition to aggregate degradation itself, the decline of biopore networks is another important component of soil structural degradation in intensified paddy fields. Biopores and the reuse of root channels have been identified as important mechanisms by which subsequent crop roots penetrate compacted layers [59]. Roots of subsequent crops often extend downward along existing cracks and old root channels, indicating a clear path-dependent effect of biopores during root re-penetration [60]. Martlew et al. found that combining cover crops with reduced tillage significantly decreased subsoil penetration resistance, and that root-derived biopores enhanced water and gas transport within compacted layers while improving the ability of subsequent crops to exploit deeper soil resources [61].

**Figure 3:**
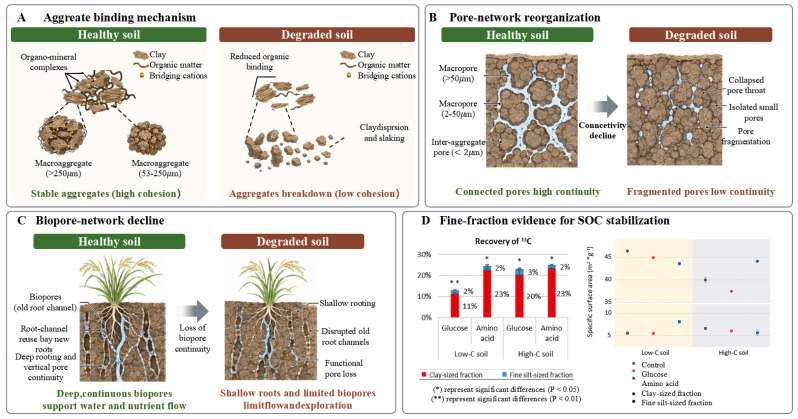
Mechanistic linkage between aggregate disruption and biopore-network decline in intensified paddy soils (**A**) Weakening of organo-mineral binding under reduced organic inputs and mechanical disturbance. (**B**) Reorganization of the pore network from connected pores to fragmented and poorly connected pores. (**C**) Decline of biopore continuity and root-channel reuse under repeated puddling and wet-soil disturbance. (**D**) Evidence for preferential carbon retention in fine mineral fractions and a higher specific surface area of clay-sized fractions. Data in Panel (**D**) were redrawn from Wu et al. [54].

As illustrated in Figure 3, beyond their role as passive indicators of structural constraints, roots actively participate in pore-network regeneration. Root penetration can reopen compacted or disconnected domains, root turnover leaves behind biopores that function as preferential pathways, and rhizodeposition supplies carbon substrates that stimulate microbial binding processes in the rhizosphere. Therefore, the loss of biopore networks represents not only a decline in physical porosity but also a weakening of root-mediated structural self-repair. Under long-term high-frequency puddling, shallow-rooting cultivation, and wet-soil mechanical disturbance, paddy soils may therefore lose not only part of their structural macropores but also the functional pore networks maintained by continuous root activity. Therefore, the diagnosis of paddy soil structural degradation should integrate aggregate stability, biopore continuity, and their functional implications for soil processes.

### 2.3. Changes in Paddy Water Regimes and Imbalance of Structural Functions

The evolution of paddy soil structure is closely coupled with water-regime dynamics. Paddy soils undergo repeated cycles of flooding, drying, re-irrigation, and drainage regulation, during which pore water occupancy, oxygen diffusion conditions, and the rhizosphere environment remain in constant adjustment [62,63]. Accordingly, changes in water regimes are not merely shifts in irrigation methods; they continuously reshape the structural functions of the plough layer and subsoil by affecting infiltration, percolation, lateral seepage, and pore aeration processes [64]. Under intermittent irrigation, controlled irrigation and drainage, and alternate wetting and drying (AWD), paddy soils gradually shift from continuous saturation to periodic unsaturation, thereby enhancing infiltration, vertical percolation, and lateral flow processes [65,66]. Intermittent irrigation can substantially influence infiltration characteristics in paddy fields, and preferential flow may also occur in lowland paddy soils [67,68,69]. These findings indicate that when paddy fields shift from prolonged ponding to wet–dry alternation, the previously stable surface water-retention state is disrupted, and water movement changes from being dominated by surface storage to enhanced vertical and lateral transport, thereby altering the hydrological response basis of the plough-layer structure.

Changes in water regimes further affect soil structural functions by regulating pore water occupancy and aeration conditions. Under long-term flooding, water-filled pores dominate, and oxygen diffusion is restricted, so the soil primarily functions in water retention and the maintenance of a puddled surface layer. Under wet–dry alternation, some pores are periodically drained, air re-enters the soil matrix, and aeration and transport functions are partially restored [63,70]. When such changes remain within an appropriate range, they can improve aeration conditions and the rhizosphere environment. However, overly rapid or intense water fluctuations may induce local crack development, excessive formation of connected pores, and a decline in structural stability, ultimately causing an imbalance in plough-layer structural functions.

In addition, adjustments in irrigation and drainage practices not only modify the water supply but also alter the migration and redistribution of nitrogen, phosphorus, potassium, and other nutrients within the soil profile [71,72,73]. Irrigation management can influence soil nitrogen supply and its vertical distribution in paddy fields, while controlled irrigation and drainage can significantly modify nitrogen leaching and lateral seepage losses [74,75,76]. HYDRUS simulations and field measurements further indicate marked differences in percolation and nutrient leaching processes under different water management regimes [77,78]. This suggests that once pore connectivity is enhanced while structural stability remains insufficient, water management adjustments may not necessarily result in structural improvement. Instead, they may accelerate nutrient loss, leading to reduced nutrient-retention capacity and unstable rhizosphere resource supply [79]. It should also be noted that appropriate water regulation may, to some extent, alleviate structural constraints and promote positive rhizosphere responses in rice [80]. Under controlled irrigation, biochar application can mitigate some adverse effects of structural degradation by improving the micropore network and local structural conditions [81,82]. The effects of water-regime changes on paddy soil structural functions are therefore dual and context dependent. Moderate wet–dry alternation may improve aeration and root-zone activity, but excessive drying or poorly synchronized nitrogen supply can intensify nutrient loss and N_2_O emissions. This duality highlights the need to evaluate water management not only by water saving or structural improvement, but also by its consequences for rhizosphere resource stability and greenhouse gas balance [83,84].

Although soil structural degradation in intensified paddy fields is often observed in the field as a combination of surface hardening, waterlogging, shallow root distribution, and poor aeration, different constraints differ in their occurrence depth, dominant structural characteristics, and primary functional limitations. Paddy soil structural degradation is therefore not a single type of constraint but can be typologically identified according to the key affected soil layer, dominant structural indicators, and the direction of functional limitation [85,86]. Under climate change, more frequent extreme rainfall, drought events, and intensified wet–dry fluctuations may further amplify these structural constraints by altering soil water status, trafficability, redox dynamics, and root-zone stability.

## 3. Typological Classification and Functional Impacts of Paddy Soil Structural Degradation

### 3.1. Typological Basis for Soil Structural Degradation in Intensified Paddy Fields

Previous studies have shown that tillage intensity, wet-soil traffic, organic matter management, and water-regime regulation can induce distinct responses in aggregate stability, pore-network continuity, hydraulic properties, and root-zone conditions [87,88]. These responses indicate that paddy soil structural degradation should not be treated as a single uniform process. In this review, we adopt a dominant-constraint-based classification and distinguish three major degradation types: compaction-dominated degradation, structure-dispersion-dominated degradation, and compound degradation. This typology is based on the dominant drivers, affected soil layers, structural symptoms, functional constraints, and field-operational red flags of degradation, as summarized in Table 1. These indicators should be interpreted as field-operational warning signs rather than universal thresholds because critical values vary with soil texture, clay content, organic matter status, rice cultivar, and management history.

Compaction-dominated degradation is mainly associated with repeated wet-soil traffic, puddling, and harvest-induced loading. Its key feature is the development of a dense subsurface layer or plough pan, which restricts percolation, aeration, and root penetration [97,98]. Structure-dispersion-dominated degradation mainly occurs when long-term straw removal, insufficient organic fertilization, or weak biological binding reduces aggregate cohesion. This type is characterized by surface sealing, unstable aggregates, weakened SOC protection, and reduced biopore continuity [99]. Compound degradation represents the coexistence of surface structural dispersion and subsurface compaction. It is more likely to occur in fields where intensive machinery operations, insufficient organic matter input, simplified cropping systems, and inadequate biological regulation occur simultaneously [100]. Unlike the first two types, compound degradation produces coupled constraints, including root-nutrient spatial mismatch, water–air imbalance, yield instability, and greenhouse gas trade-offs.

### 3.2. Functional Pathways of Different Degradation Types in Paddy Soils

The functional effects of paddy soil structural degradation are not limited to changes in individual indicators such as bulk density, aggregate abundance, or total porosity. Structural degradation alters productivity, nutrient-use efficiency, greenhouse gas emissions, and system stability by reshaping rooting space, water transport, gas diffusion, carbon and nitrogen transformations, and microbial habitats [91,93]. However, the dominant functional pathway differs among degradation types.

#### 3.2.1. Compaction-Dominated Degradation

**Figure 4:**
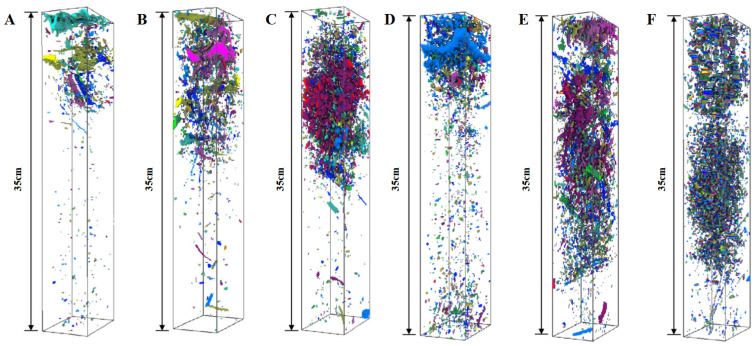
Three-dimensional structure of macropores in terraced paddy soils at different elevations. (**A**) Low elevation, inner field; (**B**) middle elevation, inner field; (**C**) high elevation, inner field; (**D**) low elevation, ridge; (**E**) middle elevation, ridge; and (**F**) high elevation, ridge. Redrawn from Ma et al. [104].

For compaction-dominated degradation, the primary constraint is mechanical impedance. Increased machinery weight and repeated wet-soil traffic intensify subsoil compaction, increase penetration resistance, and reduce connected macropores [100,101]. These changes restrict root penetration and elongation, limit deep nutrient uptake, reduce water-gas exchange, and increase the risk of runoff and nutrient depletion when surface hardening is also present [89,102]. X-ray computed tomography studies further show that plough pans, root channels, horizontal cracks, and structural pores are spatially heterogeneous in paddy soil profiles, and that tillage history can alter compacted-layer depth and bulk-density distribution in Figure 4 [103,104]. Although chiseling or subsoiling can rapidly reduce penetration resistance, newly formed pores may collapse or become blocked under rainfall, irrigation, and renewed traffic, weakening the persistence of improvements in infiltration and root growth [105]. Thus, compaction-dominated degradation should be diagnosed not only by high penetration resistance or bulk density, but also by whether the compacted layer disrupts vertical root extension, deep nutrient uptake, and water-gas exchange across the plough layer.

#### 3.2.2. Structure-Dispersion-Dominated Degradation

Structure-dispersion-dominated degradation is closely related to long-term straw removal, insufficient organic fertilization, and a lack of green manure incorporation. These practices reduce the supply of organic binding agents, making surface soil more susceptible to puddling-induced dispersion, surface sealing, and rehardening after drainage and wet–dry alternation [106,107]. The most immediate manifestation of this degradation type is the decline in surface aggregate stability [108], but it also involves an increased risk that organic carbon is transferred from physically protected pools to more labile fractions. Rhizosphere processes are particularly sensitive to structure-dispersion-dominated degradation because unstable aggregates and weakened organic carbon protection can alter rhizosphere C-N cycling, microbial habitat continuity, and root–microbe interactions. Tillage combined with straw return can increase rhizosphere acid-hydrolysable nitrogen fractions, nitrogen mineralization rates, and extracellular enzyme activities; rhizosphere organic carbon, amino sugar nitrogen, amino acid nitrogen, and related hydrolytic enzyme activities are closely associated with nitrogen mineralization processes [109]. Across different soil layers, bacterial biosynthesis, degradation functions, and genes related to the reductive tricarboxylic acid cycle contribute differently to SOC accumulation, suggesting that the effects of structural degradation on carbon cycling vary with soil depth and microbial functional traits [110]. These findings indicate that structure-dispersion-dominated degradation is primarily a problem of insufficient structural cohesion and weakened biochemical protection, with consequences extending from aggregate stability to rhizosphere C-N cycling and root–microbe interactions.

#### 3.2.3. Compound Degradation

Compound degradation combines an unstable surface structure with subsurface compaction and is therefore one of the most difficult structural constraints to remediate in intensified paddy fields. Surface aggregate instability reduces infiltration stability and resistance to structural breakdown, whereas subsurface compaction restricts vertical water transport and root penetration. Together, these processes create a water–air coupling imbalance and intensify the root-nutrient spatial mismatch within the plough layer. In compound-degraded paddy fields, yield limitation does not necessarily arise from insufficient total nutrient availability; rather, it may result from a spatial mismatch between root distribution and nutrient distribution [94,95,96]. Therefore, the restoration of compound-degraded paddy fields should not be evaluated using a single objective, such as water saving, yield improvement, or emission reduction; instead, it should consider whether the intervention can improve root distribution, stabilize nutrient acquisition, and maintain yield stability under water–air imbalance. In field diagnosis, this type should therefore be treated as a coupled structural-functional syndrome rather than as the simple coexistence of surface sealing and subsoil compaction.

## 4. Targeted Restoration Strategies for Soil Structural Degradation in Intensified Paddy Fields

Soil structural degradation in paddy fields often exhibits distinct characteristics across degradation types; therefore, a single restoration strategy is unlikely to be applicable to all degraded conditions. Effective restoration requires targeted interventions based on the dominant degradation drivers. Different restoration strategies operate through distinct mechanisms: physical regulation directly modifies soil porosity and compactness through external mechanical forces; organic fertilization promotes aggregate binding mainly through biogeochemical processes; and system-level regulation facilitates the coordinated recovery of soil structure and overall functions through multi-factor interactions.

### 4.1. Mechanical Loosening and Operational Regulation for Compaction-Dominated Degradation

For compaction-dominated degradation, the immediate restoration target is to disrupt the plough pan, reduce penetration resistance, and restore connected macropores for water percolation, gas exchange, and deep root growth. Subsoiling, deep tillage, shallow ploughing, and subsoil tillage can reduce mechanical impedance in the lower plough layer and improve porosity, hydraulic conductivity, and aeration in the middle and lower soil layers [111,112,113]. For example, shallow ploughing combined with straw return reduced soil bulk density in the 0.1–0.2 m layer by 13% compared with rotary tillage, while increasing available phosphorus, available potassium, and rice yield [114]. Deep tillage combined with organic carbon amendments can further improve the persistence of structural recovery; deep tillage to 25–28 cm with straw biochar return improved root distribution and nitrogen availability in the 7–28 cm soil layer and increased yield by enhancing panicle number and grains per panicle [93]. In paddy-upland rotation systems, intermittent deep tillage also increased the macroaggregate proportion and SOC content in the 10–30 cm layer, promoted root extension into deeper soil, and improved crop yield and economic benefits [115].

**Figure 5:**
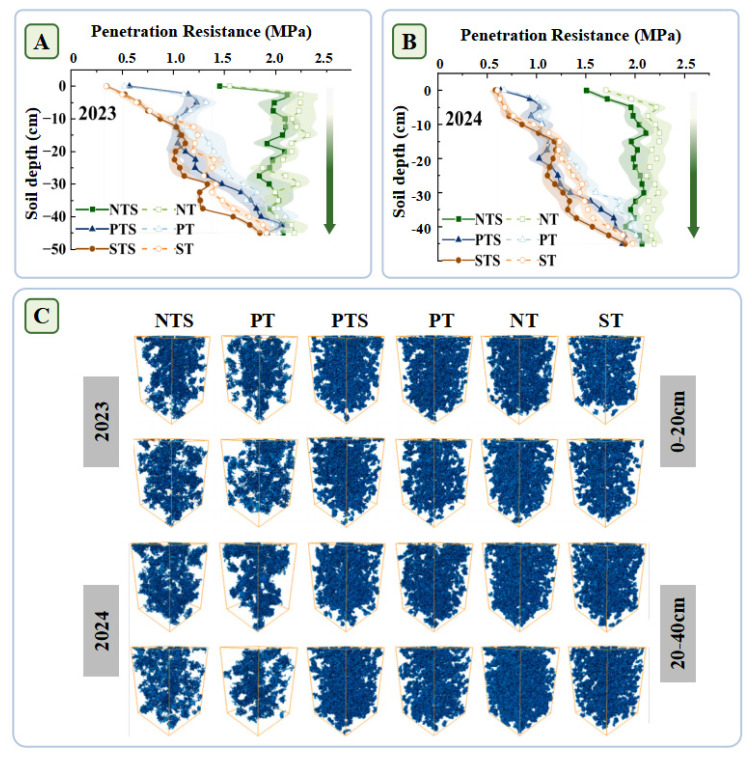
Effects of mechanical loosening and straw management on penetration resistance and aggregate pore microstructure. (**A**) Penetration resistance profiles in the 0–50 cm soil layer under different tillage and straw-management treatments in 2023. (**B**) Penetration resistance profiles in the 0–50 cm soil layer under different tillage and straw-management treatments in 2024. (**C**) Three-dimensional visualizations of aggregate pore microstructure in the −20 cm and 20–40 cm soil layers under different treatments in 2023 and 2024. This figure was redrawn and synthesized based on Sun et al. [57].

However, mechanical loosening should not be interpreted as a self-sustaining restoration measure. Its effects are often rapid but transient because newly formed macropores may collapse or become blocked under rainfall, irrigation, and renewed wet-soil traffic. The persistence of mechanical restoration depends on whether the loosened pore skeleton is stabilized by organic inputs, root growth, and controlled traffic. Rotational deep and shallow tillage can reduce the risk of long-term single-depth disturbance while periodically alleviating plough pan compaction [116,117]. Sun et al. showed that no-tillage, plough tillage, and subsoiling combined with straw return or removal produced contrasting effects on soil compaction in Northeast China. Plough tillage and subsoiling reduced penetration resistance, and subsoiling combined with straw return maintained relatively low resistance in 2024 (Figure 5A,B). More importantly, mechanical loosening combined with straw input improved aggregate pore microstructure, indicating that physical disruption of compacted layers needs to be coupled with carbon input to promote pore-structure reconstruction (Figure 5C) [57]. This evidence suggests that the key question is not whether subsoiling can temporarily reduce resistance, but whether it can create a stable pore framework that supports deeper rooting and nutrient acquisition.

Operational regulation is therefore as important as tillage itself. Timely field operations, reduced repeated traffic, controlled traffic routes, and avoidance of heavy machinery under high soil water content can reduce the formation of new compacted layers. Subsoil tillage combined with straw, organic fertilizer, or straw plus organic fertilizer has been shown to alleviate compaction and promote root length density and nutrient uptake in the 15–30 cm soil layer in rice–wheat systems [118]. For smallholder-dominated regions, however, intensive subsoiling may be limited by machinery availability, fuel costs, and field fragmentation. Thus, mechanical loosening is most suitable when applied periodically, targeted to diagnosed compacted layers, and combined with organic inputs and traffic control, rather than used as a routine annual operation.

### 4.2. Organic Fertilization and Aggregate Reconstruction for Structure-Dispersion-Dominated Degradation

**Figure 6:**
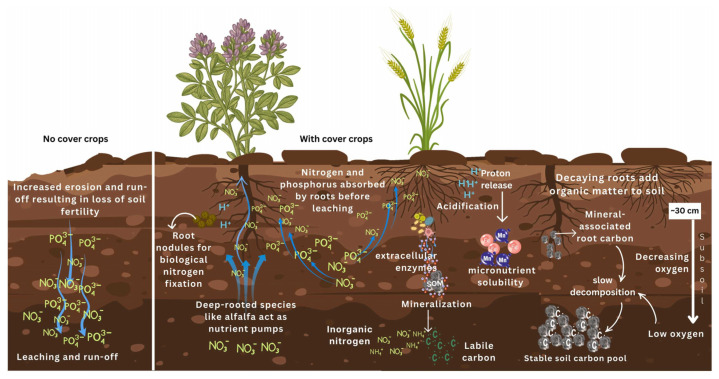
Schematic illustration of how cover crop roots and residue-derived organic inputs regulate soil chemical properties [121].

For structure-dispersion-dominated degradation, the central restoration target is to rebuild aggregate cohesion and restore biologically mediated pore stability. Straw, organic manure, green manure, and biochar can all provide exogenous carbon, but they differ in decomposition rate, binding capacity, microbial stimulation, and environmental risk [119,120]. Cover crop roots and residue-derived organic inputs regulate soil chemical processes by reducing nutrient losses, promoting nutrient cycling, and enhancing carbon sequestration, thereby supporting long-term soil fertility and environmentally sustainable nutrient management (Figure 6) [121]. By comparing straw, manure, and biochar as different residue forms, Chen et al. found that all three inputs altered the distribution of SOC pools and the molecular composition of organic matter in the paddy topsoil. Among them, biochar significantly increased SOC content and protected plant-derived carbon by promoting macroaggregate formation [122]. Manure or straw combined with chemical fertilizer can also increase the mass and carbon concentration of small macroaggregates, which are important carriers of carbon storage in paddy soils [99,123].

The critical point, however, is that organic amendments are not universally beneficial under all paddy conditions. Straw return can increase carbon input and promote aggregate formation, but fresh straw also supplies labile carbon substrates for methanogens under prolonged flooding, potentially increasing CH_4_ emissions. Organic manure can enhance aggregate binding and nutrient supply, but excessive or poorly timed application may increase nutrient losses and alter N_2_O emissions. Green manure improves SOC and dissolved organic carbon quality, but its effects depend on crop calendar, decomposition synchrony, and regional temperature-water conditions [124,125]. So organic fertilization should be viewed as a conditional restoration pathway: it is effective when carbon inputs are synchronized with water regimes, nitrogen supply, and root growth, but it may generate environmental trade-offs when used without threshold-based management.

Biochar occupies a distinct position among organic amendments because of its persistent porous structure, high stability, and potential to modify water retention, nutrient retention, and microbial habitats [126,127]. Continuous organic return and biochar amendment can reduce bulk density, enhance pore connectivity, and alleviate poor water–gas transport associated with structural dispersion [128]. Biochar may also promote macroaggregate formation through direct physical binding, electrostatic interactions, and microbially mediated binding processes [82,129]. Nevertheless, its performance is strongly dependent on feedstock type, pyrolysis conditions, application rate, soil background, and economic feasibility. Recent research on phosphorus-modified biochar prepared from Salvia miltiorrhiza residues shows that waste-derived modified biochar can immobilize Pb and Cd while contributing to resource recycling and soil fertility improvement [130]. Although this study was not conducted specifically for paddy soil structural restoration, it provides a useful example of how biochar research is moving from “soil amendment effectiveness” toward combined resource utilization, environmental remediation, and practical feasibility. For paddy systems, future work should determine whether locally available straw, manure, green waste, or medicinal residues can be converted into cost-effective biochar products that improve aggregate stability without increasing greenhouse gas risks.

### 4.3. Synergistic Restoration and System-Level Regulation for Compound Degradation

**Figure 7:**
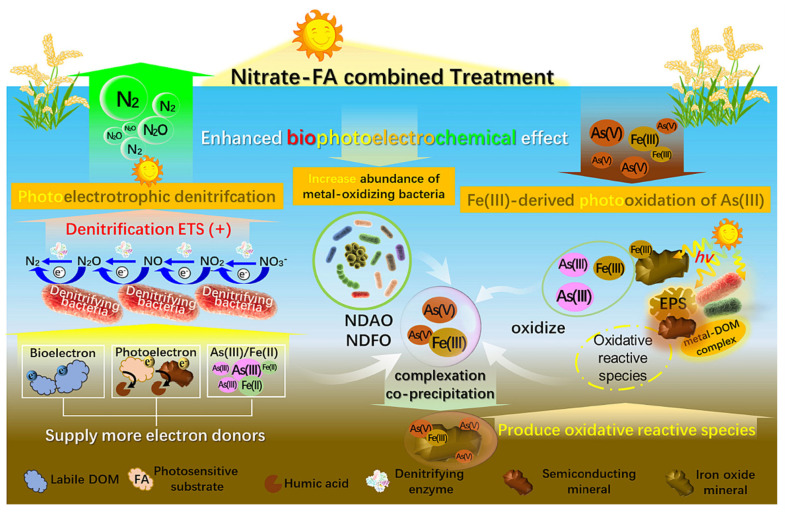
Proposed mechanisms for enhanced denitrification coupled with As(III) immobilization in illuminated flooded paddy soils amended with nitrate + FA [137].

Compound-degraded paddy fields contain both unstable surface structure and subsurface compaction; therefore, single measures usually improve only part of the constraint. Mechanical loosening can break subsurface resistance but cannot maintain pore stability alone. Organic fertilization can promote aggregate formation but has limited ability to directly disrupt compacted layers. Water-regime regulation can improve aeration and water saving but may introduce SOC loss or N_2_O emission risks if wet–dry cycles are not well controlled [131,132]. The need for this integrated approach is particularly evident in greenhouse gas trade-offs. Wu et al. reported that mild AWD combined with straw return reduced integrated global warming potential and maintained yield, but increased N_2_O emissions, with urea application being the dominant factor controlling N_2_O release [133,134,135]. Shen et al. found that AWD combined with 2% biochar reduced N_2_O emissions and increased rice yield by increasing nosZ gene copy numbers and reducing the ratios of denitrification-and ammonia-oxidation-related genes to nosZ [136]. These findings indicate that water management interacts strongly with carbon source and nitrogen transformation pathways. As shown in Figure 7, Zeng et al. further showed that fulvic acid and nitrate can stimulate denitrification and Fe-mediated redox reactions in flooded paddy soils, highlighting the importance of carbon availability, nitrate supply, and redox-active minerals in regulating microbial nitrogen pathways [137].

Subsoil carbon placement provides another example of synergistic restoration. Kan et al. proposed a ditch-buried straw return strategy that injects straw into the subsoil through deep tillage. Compared with conventional rotary-tillage straw return, this approach increased grain yield by 15%, mainly by promoting the transformation of straw-derived carbon into mineral-associated fungal necromass carbon [138]. In compound-degraded paddy fields, intermittent subsoiling can break the plough pan, organic material inputs can replenish binding agents and promote aggregate reconstruction, and optimized AWD can regulate pore aeration and redox dynamics. The restoration objective should therefore shift from maximizing a single outcome, such as yield or water saving, to balancing soil structure, root-zone function, SOC stabilization, and greenhouse gas emissions [139,140]. Another study on AWD and biochar showed that biochar can enhance SOC sequestration and reduce net GWP under AWD conditions, while suppressing AWD-induced increases in N_2_O emissions [141,142]. This suggests that biochar may serve as a complementary amendment to water regulation for mitigating nitrogen-transformation risks associated with wet–dry alternation. Long-term paddy rotation studies have also shown that rice-oilseed rape and rice-rice-oilseed rape rotations can increase topsoil porosity, total nitrogen, available phosphorus, organic matter, and aggregate composition, while enhancing microbial diversity and the complexity of community co-occurrence networks [143]. In rice-based cropping systems, zero-till transplanted rice or direct-seeded rice combined with zero tillage and residue retention in subsequent crops can increase water-stable macroaggregates, organic carbon fractions, available nutrients, and crop productivity [144].

**Figure 8:**
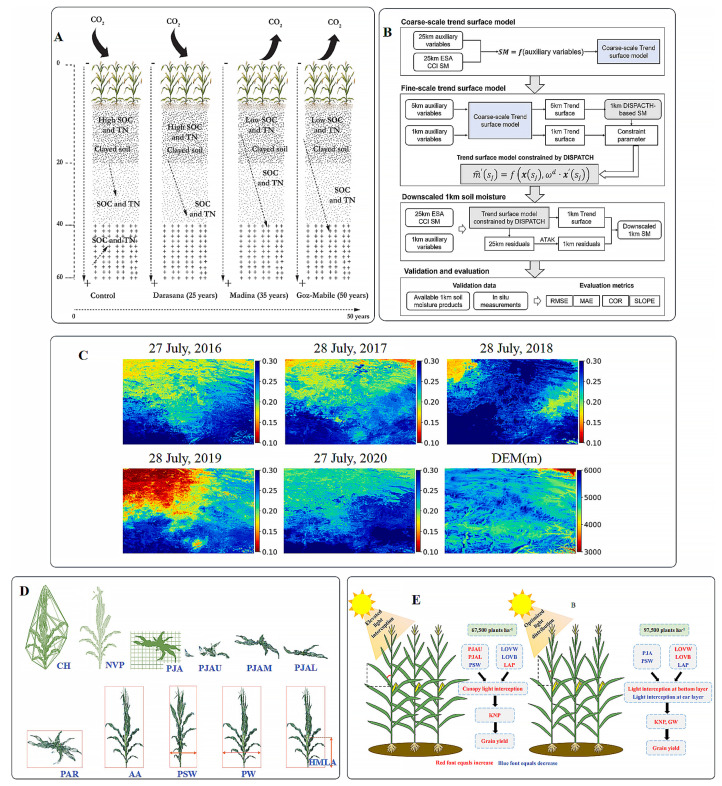
Integrated adaptive management framework for restoring compound-degraded paddy soils. (**A**) Effects of cultivation duration on SOC, TN, and CO_2_ emissions. (**B**) A physics-guided machine learning framework for soil-moisture downscaling. (**C**) Spatial heterogeneity of 1 km downscaled soil moisture. (**D**) Extraction of three-dimensional crop phenotypic traits. (**E**) Optimization of crop architecture and canopy light interception under medium and high planting densities. Panel (**A**) was redrawn from Wavel et al. [38]; panels (**B**,**C**) were redrawn from Zhang et al. [145]; panels (**D**,**E**) were redrawn from Wang et al. [146].

At larger scales, adaptive management also requires tools that can monitor soil water heterogeneity and guide irrigation thresholds. As cultivation duration increases, the risks of declining soil carbon and nitrogen stocks and increasing CO_2_ emissions become more pronounced, suggesting that the restoration of compound-degraded soils should not focus solely on the immediate effect of structural loosening but should also emphasize carbon–nitrogen maintenance and environmental-effect regulation over multi-year timescales (Figure 8A) [38,39]. Zhang et al. proposed a physics-constrained machine learning downscaling framework that can downscale coarse-resolution remotely sensed soil-moisture products to 1 km resolution. By integrating topography, vegetation, climate, soil properties, and DISPATCH-based physical constraints, this framework improves the spatial detail and physical consistency of soil-moisture estimation (Figure 8B,C) [145]. In addition to soil-structure restoration and water regulation, crop population-structure optimization may serve as a conceptual example for system-level regulation. Wang et al. used multi-view three-dimensional phenotyping to extract maize plant-architecture parameters and showed that 3D traits can characterize plant spatial occupation, canopy light interception, and their relationships with yield formation (Figure 8D,E) [146]. Although this example comes from maize rather than rice, it illustrates how crop architecture and population design can be incorporated into broader plant–soil management frameworks.

### 4.4. Comparative Mechanisms, Trade-Offs, and Restoration Risks

Different restoration strategies vary in response speed, target soil layer, persistence, and risk profile. Mechanical loosening has the fastest effect and directly targets subsurface compaction, but its persistence depends on traffic control, soil water status, and organic stabilization. Straw return, organic manure, and green manure act more slowly but can rebuild aggregate cohesion and SOC protection. Biochar has stronger persistence and pore-regulating potential, but its effectiveness is constrained by material properties, application rate, cost, and long-term field performance. Water regulation is flexible and can reduce CH_4_ emissions by improving aeration, but inappropriate drying thresholds or nitrogen timing may increase N_2_O emissions [147]. Thus, restoration should be evaluated through a “structure-yield-GHG” framework rather than through a single agronomic or soil physical indicator. The target objects, applicable conditions, and potential risks of paddy soil structural restoration strategies are summarized in Table 2.

From a mechanistic perspective, restoration can be interpreted at three levels. First, physical loosening directly reconstructs the pore skeleton and creates space for water movement and root penetration. Second, organic inputs provide binding agents and carbon substrates that stabilize aggregates and support microbial-mediated structural recovery. Third, water regulation and cropping-system design determine whether the restored pore system remains aerated, biologically active, and environmentally sustainable. Through these coupled processes, degraded paddy soils can be restored in a more persistent and functionally coordinated manner [156,157].

### 4.5. Practical Applicability and Adoption Constraints

The field adoption of restoration practices depends not only on their biophysical effectiveness but also on cost, labour demand, machinery accessibility, residue availability, and management complexity. Subsoiling is effective for breaking compacted layers, but it requires specialized machinery, fuel inputs, and suitable soil water conditions. These requirements may limit its feasibility in fragmented smallholder landscapes or regions where machinery services are not readily available. Straw return and organic manure application are often more accessible, but their effectiveness depends on residue collection, transportation, labour organization, decomposition timing, and water management. Inappropriate straw incorporation may also increase CH_4_ emissions under continuous flooding. Waste-derived modified biochar, such as phosphorus-modified biochar produced from medicinal plant residues, provides a promising direction because it links residue recycling, soil fertility improvement, and environmental remediation. Nevertheless, its agronomic return, cost–benefit ratio, and long-term effects on paddy soil structure and greenhouse gas balance require field-scale validation. For practical restoration, the most feasible pathway may not be the most technically intensive measure, but a locally adapted combination of moderate mechanical intervention, available organic resources, threshold-based irrigation, and rotation design.

In this context, restoration recommendations should be differentiated by production scale. Large-scale mechanized farms may prioritize controlled traffic, periodic subsoiling, precision irrigation, and integrated residue management. Smallholder systems may benefit more from service-based mechanization, low-cost organic inputs, green manure, optimized straw handling, and water-saving practices that do not require expensive equipment. Therefore, paddy soil structural restoration should be developed not only as a scientific framework but also as a decision-making system that accounts for local resources, labour, machinery access, and farmer adoption capacity.

## 5. Self-Recovery Mechanisms and System Physical Resilience of Restored Paddy Soil

A stable restoration effect in paddy soil structure should not be evaluated only by short-term reductions in bulk density or penetration resistance. More importantly, restored soils should be able to maintain water transport, gas exchange, aggregate stability, root-zone functionality, and carbon–nitrogen cycling under alternate wetting and drying, root growth, organic carbon inputs, microbial activity, and mild mechanical disturbance. System physical resilience therefore refers to the capacity of the soil to maintain and recover structural functions after disturbance. This capacity depends on the coupled interactions among pore networks, aggregates, organic carbon fractions, rice root activity, rhizosphere processes, and microbial functional groups [158,159].

### 5.1. Persistent Stabilization of Pore Networks and Aggregate Structure

Moderate water regulation can facilitate the transition of the pore system from a long-term water-filled state to a dynamic state characterized by alternating water and gas transport, thereby improving the rhizosphere pore environment and facilitating root-zone water uptake. Using X-ray computed tomography, a miniaturized infiltrometer, and small-scale indentation tests, Islam et al. found that, under AWD, root-zone macroporosity increased by 46%, pore connectivity increased by 20%, and root-zone water absorption rate increased by approximately 36% compared with continuous flooding [81]. This evidence indicates that appropriate water regulation can improve not only soil hydraulic behaviour but also the physical environment directly experienced by rice roots.

The persistence of these improvements depends on whether the newly formed or reconnected pores can be stabilized by organic carbon and aggregate protection. Long-term organic manure application increases SOC in paddy soils and promotes SOC storage in slow-cycling fractions, such as intra-microaggregate particulate organic matter and free silt-clay fractions [160]. Under comparable carbon inputs, paddy soils may exhibit a relatively high SOC retention potential, probably because of lower microbial decomposition, Fe-related chemical stabilization, and physical protection within aggregates [161]. Based on synchrotron-based X-ray micro-computed tomography, phospholipid fatty acid analysis, and SOC density fractionation, Zhang et al. found that subsoiling combined with straw return not only improved aggregate micropore structure but also enhanced microbial biomass and promoted SOC accumulation; notably, microbial characteristics explained more variation in intra-aggregate SOC than pore-structure characteristics [162]. These findings indicate that structural recovery is not merely a physical rearrangement of particles and pores. Rather, persistent recovery requires the co-development of pore formation, aggregate binding, organic carbon protection, and microbial stabilization.

### 5.2. Rice Root Traits and Rhizosphere Processes as Active Drivers of Soil Structural Rehabilitation

Rice roots represent a biological engine for structural self-recovery because they continuously reshape rhizosphere pores, redistribute organic carbon inputs, and regulate microbial habitats through penetration, exudation, senescence, and residue decomposition. Soil management strategies based on straw mulching and biochar amendment may further enhance these processes while providing environmental benefits related to climate-change mitigation [163]. Under AWD conditions, organic manure and straw treatments increased root length density and root weight density by approximately 30% and 40%, respectively, compared with chemical fertilizer alone, while also enhancing root activity, root oxidation capacity, and N, P, and K uptake [164]. Thus, appropriate water regimes and organic inputs can jointly improve root function and provide a biological driving force for structural recovery.

Organic management can also increase soil pH and available nutrients, alleviate microbial carbon and nitrogen limitations, and enhance microbial stoichiometric homeostasis and carbon use efficiency [165]. Complex crop rotations combined with full straw return can increase microbial carbon use efficiency (CUE) and microbial necromass turnover, promote the formation of mineral-associated organic carbon (MAOC), and direct more carbon inputs into stable mineral-associated carbon pools [166]. Therefore, the contribution of root–microbe processes to structural recovery is not limited to stimulating organic matter decomposition; it also involves the formation of microbial necromass and stable carbon pools. Pereira-Mora et al. showed that different rice rotation intensities significantly affected the methanogenic archaeal community structure and methane production potential, with higher mcrA gene copy numbers and relative abundances of methanogenic archaea in continuous rice systems [167]. Accordingly, root and microbial processes may promote SOC stabilization and aggregate formation, but they may also increase CH_4_ emission risks under long-term reducing conditions. Future research should therefore evaluate both the structural effects and greenhouse gas consequences of rhizosphere carbon inputs.

### 5.3. Microbial Functional Groups, Carbon–Nitrogen Cycling, and Greenhouse Gas Trade-Offs

The microbial contribution to paddy soil structural recovery should be discussed in terms of specific functional groups rather than generalized microbial activity. EPS-producing bacteria contribute to particle binding and aggregate stabilization by producing extracellular polymeric substances in the rhizosphere. Methanogenic archaea, commonly represented by the mcrA gene, drive CH_4_ production under strongly reducing conditions when labile carbon from straw, manure, or root exudates is abundant. Methanotrophs, commonly linked to pmoA, can partly offset CH_4_ production by oxidizing methane in oxygenated microsites around rice roots and drained pore domains. Nitrifiers and denitrifiers, represented by amoA, nirK, nirS, and nosZ, regulate nitrification, denitrification, N_2_O production, and N_2_O reduction under fluctuating redox conditions. Fe-reducing and Fe-oxidizing microorganisms further influence the formation and dissolution of Fe oxides, thereby affecting organo-mineral associations, SOC stabilization, and nitrogen transformation in redox-dynamic paddy environments [168].

Biochar and organic amendments can modify these microbial pathways, but their effects are not uniform. Biochar-based fertilizer has been reported to mitigate paddy soil N_2_O emissions by shifting the balance between nitrifier and denitrifier functional genes and improving nitrogen recovery efficiency [169]. However, the direction of biochar effects may depend on feedstock, pyrolysis conditions, pH, redox status, and the relative abundance of genes associated with N_2_O production and reduction. Recent evidence from flooded paddy soils further demonstrates that carbon substrates, nitrate availability, and redox-active minerals can interactively regulate denitrification and Fe-mediated redox reactions. Zeng et al. showed that illuminated fulvic acid stimulated denitrification and As(III) immobilization in flooded paddy soils through an enhanced biophotoelectrochemical pathway, emphasizing that labile carbon, nitrate, Fe cycling, and electron transfer processes can jointly regulate microbial nitrogen pathways under flooded conditions [137].

Accordingly, future evaluation of paddy soil structural rehabilitation should quantify not only SOC accumulation and aggregate stability but also microbial functional pathways. Key indicators should include EPS-producing microbial groups, mcrA for methanogenesis, pmoA for methane oxidation, amoA for nitrification, nirK and nirS for N_2_O production potential, and nosZ for N_2_O reduction capacity. Combining these functional genes with pore-network imaging, root-zone oxygen dynamics, and GHG flux measurements would allow researchers to determine whether a restoration practice truly improves plant–soil functioning or merely shifts the system from one environmental risk to another.

### 5.4. Climate Adaptability and Agroecosystem Resilience

The resilience of restored paddy soils is also reflected in their integrated responses to wet–dry fluctuations, extreme rainfall, water scarcity, and mechanized operations [170]. AWD, controlled irrigation, biochar amendment, straw return, and crop rotation can all influence soil water status, carbon–nitrogen transformations, and microbial community structure, but their effects are strongly context dependent. Optimized irrigation management can reduce irrigation water use, improve water productivity, and lower greenhouse gas emissions. However, water-saving irrigation may also increase soil–atmosphere carbon fluxes and reduce SOC and soil organic nitrogen. Therefore, AWD should be regarded as a water management strategy that requires threshold control and complementary measures, rather than as a stand-alone structural restoration practice [171]. Long-term resilience assessment should accordingly incorporate pore structure, SOC fractions, and microbial community stability. From a plant-production perspective, such resilience should ultimately be reflected in stable root activity, nutrient acquisition, and yield formation under fluctuating water and mechanical disturbance regimes.

## 6. Long-Term Maintenance Mechanisms and Adaptive Management for Global Sustainability

The long-term goal of paddy soil structural restoration is to improve water and nutrient use efficiency, promote SOC stabilization, reduce greenhouse gas emissions, and adapt to mechanized operations while maintaining grain production. Therefore, paddy soil structural restoration should shift from the evaluation of single practices toward integrated management based on degradation type, soil conditions, and production systems.

### 6.1. Long-Term Coupling of Soil Structure/Microbial Processes and Organic Carbon Stabilization

The long-term maintenance of restored paddy soil structure depends on the coupling of physical structure, organic carbon stabilization, root activity, microbial processes, and adaptive field management. Long-term cultivation and amendment studies indicate that SOC storage is closely associated with macroaggregate stability, microbial network complexity, fungal community composition, microbial carbon use efficiency, and microbial necromass formation [172,173,174]. Organic manure, straw, green manure, and biochar can contribute to structural stability through different pathways, including aggregate formation, mineral-organic association, microbial necromass accumulation, and Fe/Al/Mn oxide-mediated carbon stabilization. However, these inputs should be combined according to target functions rather than applied uniformly.

Different organic inputs operate through distinct functional pathways, and long-term management should combine them according to target functions [175]. Adaptive management should also distinguish between production systems. Flooded rice monoculture systems require sufficient water-retention capacity and stable surface ponding, whereas paddy-upland rotation systems require greater profile permeability, root-zone drainage, and resistance to recompaction. Under climate change and increasing mechanization, water scarcity, extreme rainfall, wet–dry fluctuations, and wet-soil traffic may jointly increase the risk of structural instability [176,177,178].

### 6.2. Future Outlook: From Static Diagnosis to Predictive and Site-Specific Restoration

Future research should move beyond static descriptions of paddy soil degradation toward dynamic, predictive, and site-specific restoration. X-ray CT and synchrotron micro-CT can quantify pore connectivity, macropore continuity, root-channel inheritance, and aggregate-scale recovery. Rhizosphere metabolomics and root exudate profiling can clarify how rice roots regulate microbial habitats, mucilage production, aggregate binding, and nutrient transformation. Microbial multi-omics can further identify the functional groups and genes involved in methanogenesis, methane oxidation, nitrification, denitrification, EPS production, and Fe-mediated redox processes.

At larger scales, digital soil twins and interpretable machine learning approaches may help integrate soil structure, water regime, root traits, microbial functions, management practices, and climate drivers into predictive restoration frameworks. Recent advances in AI-enabled agricultural digital twins and dual-scale interpretable machine learning for soil moisture dynamics suggest that multi-source data integration can support soil-moisture prediction, irrigation scheduling, machinery operation, and adaptive farm management [179,180]. These tools could support early warning of structural degradation, threshold-based irrigation and traffic decisions, and regional matching of restoration strategies to degradation types. Future paddy soil restoration should therefore develop from experience-based management toward diagnosis-guided, data-supported, and locally adaptable decision-making.

## 7. Conclusions

Soil structural degradation in intensified paddy fields results from the combined effects of long-term mechanical disturbance, wet-soil operations, insufficient organic carbon inputs, changes in water regimes, and simplified cropping systems. It is not manifested as a single process of surface hardening or compaction; rather, it represents a systemic structural degradation process involving subsoil densification, surface aggregate instability, reduced pore connectivity, loss of biopores, and an imbalance in water–air coupling. According to the dominant drivers, occurrence layers, and functional constraints, soil structural degradation in intensified paddy fields can be classified into three types: compaction-dominated degradation, structure-dispersion-dominated degradation, and compound degradation.

Different degradation types affect paddy soil functions through distinct pathways. Compaction-dominated degradation mainly restricts deep root penetration, water–gas transport, and the utilization of nutrients in deeper soil layers. Structure-dispersion-dominated degradation primarily weakens aggregate stability, organic carbon protection, and rhizosphere carbon–nitrogen cycling. Compound degradation is characterized by the combined effects of surface sealing, restricted subsurface water movement, abnormal nutrient transport, and trade-offs in greenhouse gas emissions. Therefore, paddy soil structural restoration must shift from conventional single-practice improvement toward typology-based and targeted restoration.

Existing evidence indicates that mechanical loosening and deep tillage can rapidly alleviate compaction constraints, but their persistence depends on the joint maintenance effects of straw return, organic manure, green manure, biochar amendment, and root-mediated processes. Organic fertilization and biochar can improve structural stability by increasing carbon inputs, promoting aggregate formation, enhancing mineral–organic associations, and improving pore networks. Water regulation, crop rotation, and conservation tillage can further influence system resilience by modifying redox conditions, root distribution, and microbial functions.

The main contribution of this review is to propose a plant–soil-centred framework for understanding and restoring paddy soil structural degradation. This framework links degradation typology, field diagnosis, root-rhizosphere processes, microbial functional groups, greenhouse gas balance, and practical restoration pathways. Future restoration should aim not only to reduce bulk density or increase yield in the short term, but also to rebuild stable pore networks, sustain aggregate turnover, protect organic carbon, regulate microbial carbon–nitrogen pathways, and support resilient rice root growth and plant–soil feedbacks under intensified production. Integrating advanced imaging, rhizosphere metabolomics, microbial multi-omics, field GHG monitoring, digital soil twins, and machine learning will be important for developing threshold-based, site-specific, and operational restoration strategies for sustainable paddy systems.

## Figures and Tables

**Table 1:** Typology, diagnostic features, and field-operational red flags of soil structural degradation in intensified paddy fields.

Degradation Type	Drivers	Structural Symptoms	Functional Constraints	Field-Operational Red Flags	Suggested Diagnostic Indicators	Ref.
Compaction-dominated	Wet-soil traffic; repeated machinery operations	Dense subsoil reduced connected pores	Poor percolation, aeration, and rooting	Hard subsurface layer; slow percolation; shallow root distribution	Bulk density; penetration resistance; compacted-layer depth; root depth; macroporosity	[89,90]
Structure-dispersion-dominated	Low organic inputs; frequent puddling	Unstable aggregates; weak SOC protection	Surface sealing and unstable C-N cycling	Surface sealing; rapid rehardening after drainage; weak aggregate cohesion	Water-stable aggregates; MWD/GMD; SOC-to-clay ratio;	[91,92]
Compound degradation	Traffic pressure plus organic matter depletion	Surface dispersion with subsoil compaction	Root-nutrient mismatch and water–air imbalance	waterlogging; shallow rooting; uneven nutrient distribution	Penetration resistance; infiltration rate; root distribution; nutrient-depth profile; CH_4_/N_2_O fluxes	[93,94,95,96]

**Table 2:** Key features of restoration strategies for paddy soil structural degradation.

Strategy	Target	Core Effect	Applicable Type	Main Risk	References
Subsoiling/shallow ploughing	Plough pan; compacted subsoil	Lowers penetration resistance; improves subsoil rooting	Compaction-dominated; compound	Recompaction risk without organic inputs or traffic control	[148]
Straw return/organic manure	Surface aggregates; SOC fractions	Increases SOC and macroaggregates	Structure-dispersion-dominated; compound	Possible CH_4_ increase under flooding; method-dependent effects	[149]
Green manure/rotation	Root residues; microbial networks	Enhances aggregates and microbial diversity	Structure-dispersion-dominated; compound	Constrained by the crop calendar, climate, and rotation feasibility	[150,151,152]
Biochar amendment	Pores; SOC stability; microbial habitat	Improves pore connectivity and SOC stabilization	Structure-dispersion-dominated; compound	Cost, application rate, and long-term uncertainty	[153,154]
AWD/controlled irrigation	Pore water; redox conditions; N cycling	Saves water and reduces CH_4_ emissions	Compound; climate-adaptive	Potential N_2_O increase and SOC destabilization under inappropriate thresholds	[23,155]

## Data Availability

No new datasets were generated in this study. All information analyzed in this review was derived from publicly available literature indexed in the Web of Science Core Collection and Science Direct.
